# Haemoglobin changes and risk of anaemia following treatment for uncomplicated falciparum malaria in sub-Saharan Africa

**DOI:** 10.1186/s12879-017-2530-6

**Published:** 2017-06-23

**Authors:** Julien Zwang, Umberto D’Alessandro, Jean-Louis Ndiaye, Abdoulaye A Djimdé, Grant Dorsey, Andreas A Mårtensson, Corine Karema, Piero L. Olliaro

**Affiliations:** 1 Independent Researcher, Mae Sot, Thailand; 2 Medical Research Council Unit, Fajara, Banjul, The Gambia; 3 London School of Hygiene and Tropical Medicine, London, UK; 4 Institute of Tropical Medicine, Antwerp, Belgium; 5 Department of Parasitology, Faculty of Medicine, Cheikh Anta Diop University, Dakar, Senegal; 6 Malaria Research and Training Center, Department of Epidemiology of Parasitic Diseases, Faculty of Pharmacy, University of Science, Techniques and Technologies of Bamako, Bamako, Mali; 7 Department of Medicine, University of California San Francisco, San Francisco, California, USA; 8 Department of Microbiology, Tumor and Cell Biology, Karolinska Institutet, Stockholm, Sweden; 9 Department of Women’s and Children’s Health, International Maternal and Child Health (IMCH), Uppsala University, Uppsala, Sweden; 10 Swiss Tropical and Public Health Institute, Basel, Switzerland; 11 University of Basel, Basel, Switzerland; 12 Special Programme for Research & Training in Tropical Diseases (WHO/TDR), 20 Avenue Appia, 1211 Geneva, Switzerland; 13 Centre for Tropical Medicine and Global Health, Nuffield Department of Medicine, University of Oxford, Churchill Hospital, OX37LJ, Oxford, UK

**Keywords:** *P. Falciparum*, Malaria, Artemisinin, Anaemia, Haemolysis, Artesunate, Amodiaquine, Sub-Saharan Africa

## Abstract

**Background:**

Anaemia is common in malaria. It is important to quantitate the risk of anaemia and to distinguish factors related to the natural history of disease from potential drug toxicity.

**Methods:**

Individual-patient data analysis based on nine randomized controlled trials of treatments of uncomplicated falciparum malaria from 13 sub-Saharan African countries. Risk factors for reduced haemoglobin (Hb) concentrations and anaemia on presentation and after treatment were analysed using mixed effect models.

**Results:**

Eight thousand eight hundred ninety-seven patients (77.0% <5 years-old) followed-up through 28 days treated with artemisinin combination therapy (ACT, 90%, *n* = 7968) or non-ACT. At baseline, under 5’s had the highest risk of anaemia (77.6% vs. 32.8%) and higher parasitaemia (43,938 μl) than older subjects (2784 μl). Baseline anaemia increased the risk of parasitological recurrence.

Hb began to fall after treatment start. In under 5’s the estimated nadir was ~35 h (range 29–48), with a drop of −12.8% from baseline (from 9.8 g/dl to 8.7 g/dl, *p* = 0.001); in under 15’s, the mean Hb decline between day 0–3 was −4.7% (from 9.4 to 9.0 g/dl, *p* = 0.001). The degree of Hb loss was greater in patients with high pre-treatment Hb and parasitaemia and with slower parasite reduction rates, and was unrelated to age. Subsequently, Hb increased linearly (+0.6%/day) until day 28, to reach +13.8% compared to baseline.

Severe anaemia (<5 g/dl, 2 per 1000 patients) was transient and all patients recovered after day 14, except one case of very severe anaemia associated with parasite recurrence at day 28.

There was no systematic difference in Hb concentrations between treatments and no case of delayed anaemia.

**Conclusion:**

On presentation with acute malaria young children with high parasitaemia have the highest risk of anaemia. The majority of patients experience a drop in Hb while on treatment as early as day 1–2, followed by a linear increase through follow-up. The degree of the early Hb dip is determined by pre-treatment parasitaemia and parasite clearance rates. Hb trends and rick of anaemia are independent of treatment**.**

**Electronic supplementary material:**

The online version of this article (doi:10.1186/s12879-017-2530-6) contains supplementary material, which is available to authorized users.

## Background

As anaemia is a common occurrence in malaria, it is often difficult, in treated patients, to disentangle disease from drug effects. Malaria anaemia has both central (decreased red cell production) and peripheral causes (haemolysis, phagocytosis of infected and uninfected erythrocytes) [1, 2]. Artemisinin-based Combination Therapy (ACT) - the treatment of choice for uncomplicated *Plasmodium falciparum* malaria [3] - is generally safe and well-tolerated, but anaemia remains a potential concern, in particular for artemisinin derivatives. In vitro experiments point to interference with erythropoiesis at the proerythroblast and basophilic erythroblast stage [4]. In humans, acute delayed haemolytic anaemia has been reported following artesunate injections in severe malaria [5], but not with ACT in uncomplicated falciparum malaria; transient anaemia has been reported in uncomplicated malaria after administration of artesunate, alone or as part of ACT [6–11], with a dose-dependent risk [12]. As safety in general, and laboratory data in particular, are under-reported in antimalarial drug trials, limited information can be derived from aggregated data from the published literature. In particular anaemia, even when monitored, is variably reported as a continuous variable (haemoglobin concentration) or categorical variable (using different thresholds and severity classes).

This group therefore joined to create an individual participant-level database (IPD) of patients enrolled in studies conducted in Sub-Saharan Africa and applied a common, standardised analytical approach to quantify the risk of anaemia in patients presenting with acute, uncomplicated falciparum malaria, and analysed the changes in haemoglobin levels and anaemia status after treatment.

## Methods

Data on age, parasitaemia, haemoglobin (Hb), treatment and treatment outcome were extracted from primary data of nine randomized controlled trials (RCT) conducted in sub-Saharan Africa, of which seven had 28-day [13–20], and two had 42-day follow-up [20, 21]. The RCTs included in this paper were conducted to assess the efficacy and safety of different malaria treatment in sub-Saharan Africa using the WHO protocol (Zwang 2009, Zwang 2014). Data were censored when patients were lost to follow-up or had microscopy determined recurrent parasitaemia during follow-up.

Anaemia was defined and graded as per WHO, 2011 by age and sex [22] (Additional file 1: Table S1). Recovery from anaemia was defined as Hb increasing to levels above the aforementioned levels. Worsening of anaemia was any increase at any time in the grade of severity compared to pre-treatment (abnormal Hb in subjects with normal pre-treatment value, or an abnormal value that was of higher grade than pre-treatment). Drug-event relationship could not be attributed as it had not been assessed systematically across the clinical trials.

Treatments were artesunate-amodiaquine (ASAQ, as either fixed-dose or co-blister formulations) and comparators including artemisinin combination therapy (ACT: artemether-lumefantrine, AL; artesunate plus sulphadoxine/pyrimethamine, AS + SP; dihydroartemisinin-piperaquine, DP) or monotherapy (artesunate alone, AS; amodiaquine alone, AQ) or non-artemisinin containing combinations (AQ + SP).

### Ethics

All trials were conducted according to the Helsinki declaration and received approval by their respective ethics committees. These analyses are in accordance with the patient informed consent. Data were completely and irreversibly anonymised at the source before being shared and merged into the common database.

### Statistical analysis

Categorical data (proportions of patients becoming or recovering from anaemia) were compared using the chi-square or the Fisher’s exact test. For non-normally distributed data comparisons used the Mann-Whitney and the Kruskall-Wallis tests and correlations, the Spearman test.

Definitions: D (Day) 0 = pre-treatment; HDR_d1_ (Hb decrease rate) = percentage difference in Hb concentration from D0 to D1; PRR_d1_ (parasite reduction rate) = percentage difference in parasite density from D0 to D1; PCT (parasite clearance time) = time to reach a negative malaria smear for two consecutive days; nadir = the lowest Hb value at any time on-study; T_n_ (time-to-nadir) = time at which the nadir was reached for each study subject.

All available Hb values for every patient were considered from D0 through D28. The risk of anaemia was assessed by mixed-effect logistic regression model to account for between-sites heterogeneity. The adjusted risks (AOR) allowed for patients’ age, parasitaemia (log-transformed), parasitological recurrence, treatment and trial.

The risk of Hb dropping, resulting in anaemia at D3, or anaemia severity increasing, was analysed as a binary variable by mixed-effect logistic regression models. The percentage Hb decrease at nadir was analysed using mixed-effect linear regression models. Hb trends from D3 to D28 were assessed by a mixed-effect linear regression model and expressed as *y = ax + b* (were *y*, Hb; *a*, slope; *x*, time; *b*, constant).

Confidence intervals were calculated at 95% (95%CI), and comparisons considered significant when *p* < 0.05. Data were analysed using Stata v13 (Stata Corp.).

## Results

Analyses were based on 8897 patients, 89.6% treated with an ACT and 10.4% with non-ACTs (Additional file 2: Table S2, Additional file 3: Fig. S1), providing 38,864 Hb measurements (93% and 7% for ACT and non-ACT, respectively).

Study characteristics are in Additional file 4: Table S3: 98% (*n* = 8728) of subjects had at least one other recorded value post-D0 (58% at D3, 80% at D7, 73% at D28); 18% (*n* = 1645) children <5 years-old enrolled in Liberia, Mozambique, Rwanda and Uganda had daily measurements on D0 through D3; an additional 11% (*n* = 1000) had daily measurements on D0 through D2. All but two studies (Uganda and Rwanda) had at least three measurements: D0, D28 and one in-between: D7 (11%, in four countries), D14 (20%, two countries), and D3, D7, and D14 (17%, two countries).

**Fig. 1:**
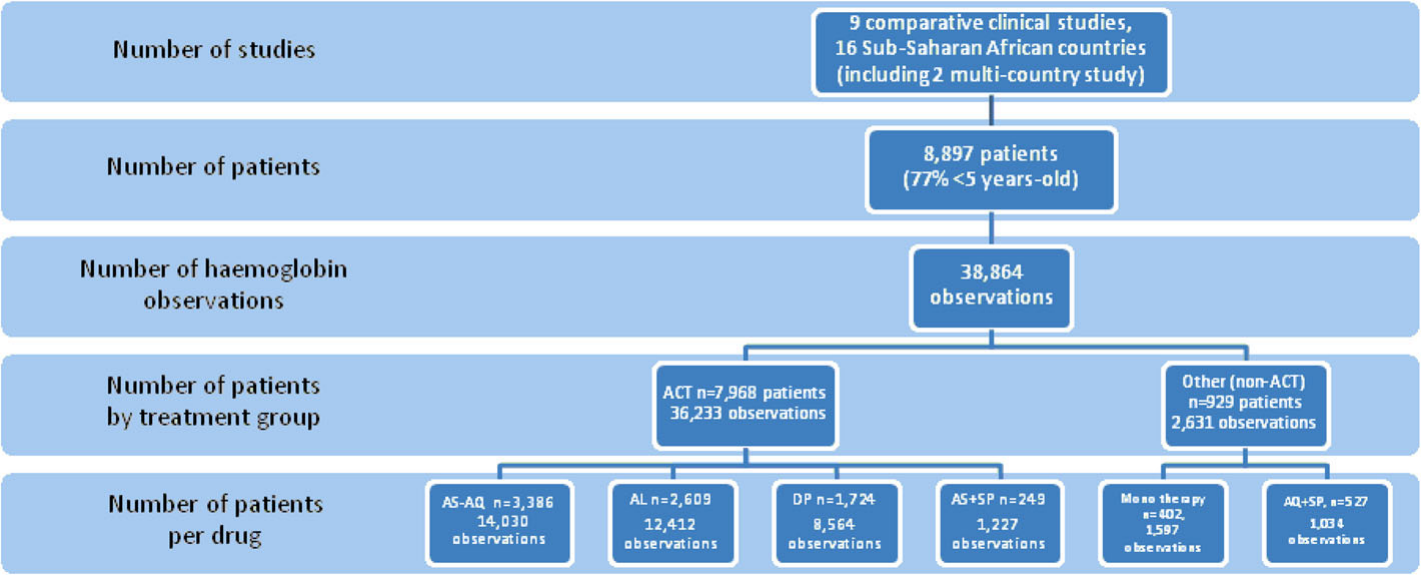
Flow chart

Patients’ baseline characteristics by study are in Table 1 and Fig. 1: 77% were children <5 years of age, 24.1% were 5–11 years-old, 4.5% were 12–14 years-old, and 11.0% ≥15. The geometric mean D0 parasite density was 13,790 overall, ranging from 594 (Liberia [19]) to 39,023 parasites/μl (Mozambique [13]).

### Anaemia and haemoglobin levels on presentation with acute falciparum malaria

Sixty-seven percent (67.3%, *n* = 5978/8897) of subjects had anaemia on D0, 63% mild or moderate. The mean D0 Hb was 10.2 g/dl (standard deviation 2.05) and the median 10.1 g/dl (range 3.4–18.3).

**Fig. 2:**
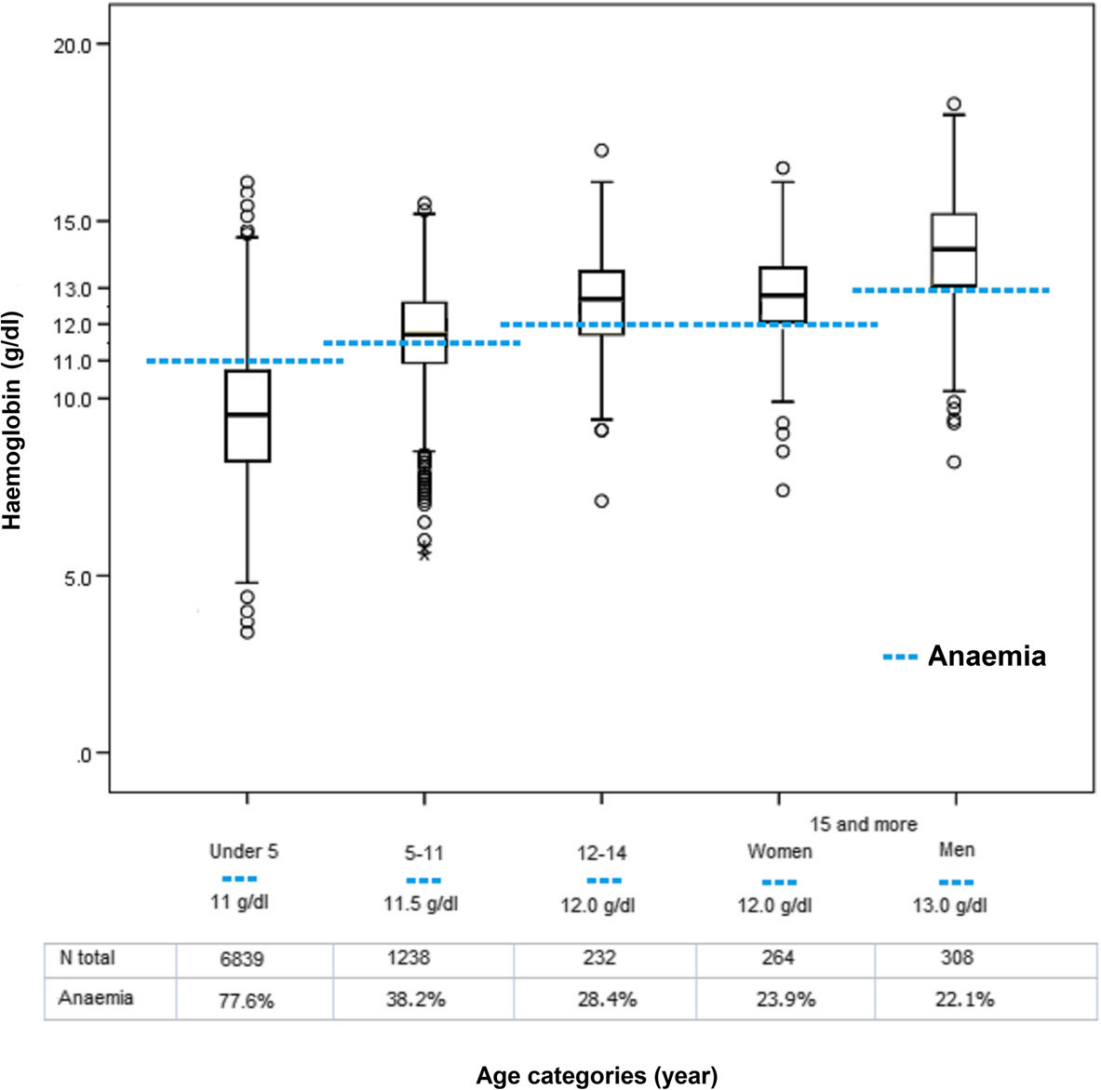
Boxplot of haemoglobin concentration values pre-treatment (D0) by age and sex category. The dotted blue line represents the anaemia-defining Hb value for each category

Hb increased with age (from a median of 9.6 g/dl in < 5 years-old to 14.1 g/dl in males ≥15 years), corresponding to a decreasing prevalence of anaemia (from 77.6% to 22.1%, respectively, Fig. 2).

A multivariate logistic regression model with mixed effects found that the risk of D0 anaemia decreased significantly with increasing age (*p* = 0.001 for all comparisons vs. under 5’s). Parasitological recurrence during follow-up was more frequent in subjects with D0 anaemia (AOR 1.45, 95% CI 1.23–1.73, *p* = 0.001).

**Fig. 3:**
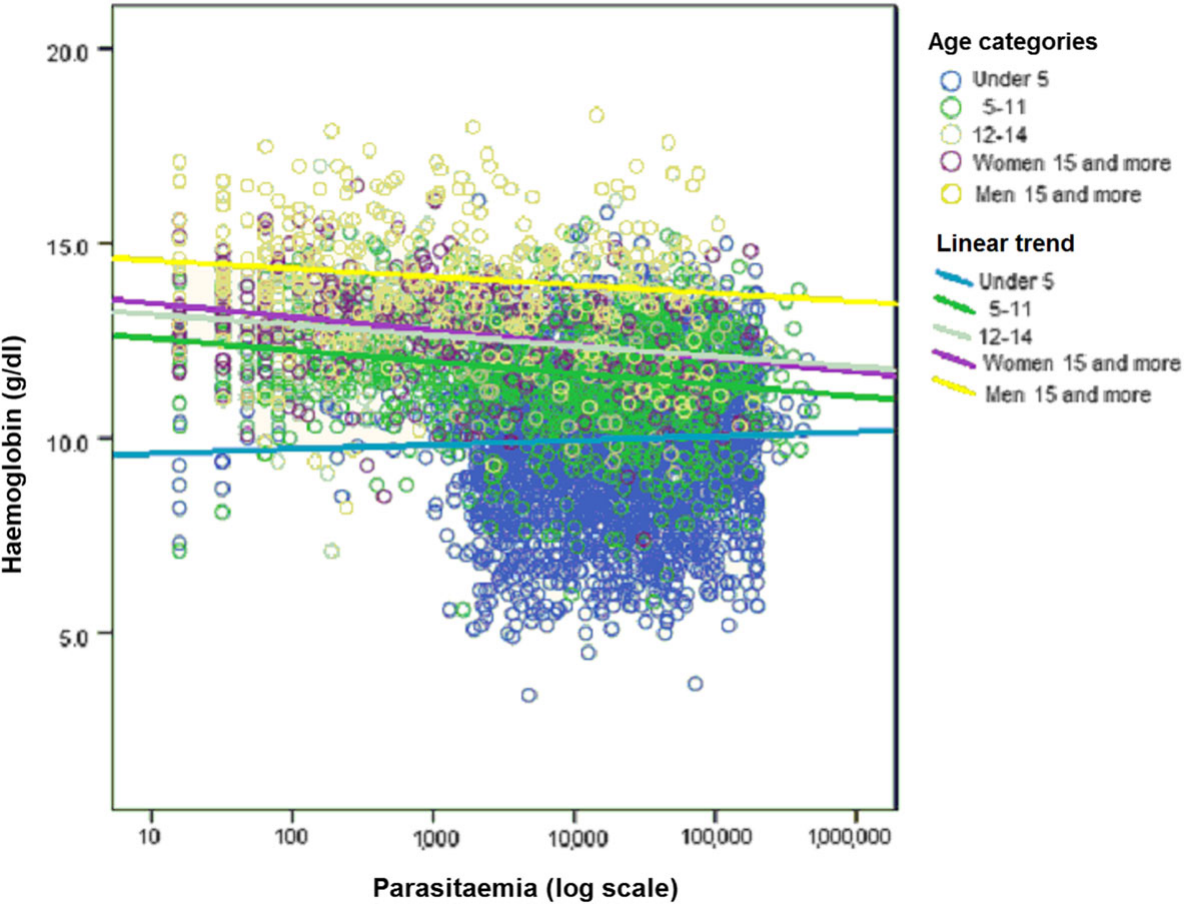
Scatter plot of haemoglobin concentration values and parasitaemia pre-treatment (D0): overall and by age and sex category. AOR for 5–11 years-old of 0.51 (95% CI 0.41–0.62); 12–14 AOR 0.41 (0.28–0.60); ≥15 AOR 0.27 (0.20–0.37). For the whole study population the equation calculated by linear regression with mixed effect model was y = − 0.06× + 10.3, *p* = 0.020

Overall, higher D0 parasite density correlated with lower Hb by linear regression, mixed effect model (Fig. 3) except for <5 year-olds who had higher D0 geometric mean parasite densities (43,938 μl) than older subjects (2784 μl) (Additional file 5: Fig. S2).

### Anaemia during treatment and follow-up

#### Occurrence of anaemia

From D0 to last observation, 82.7% of the patients had at least one Hb measurement fulfilling the anaemia criteria (any grade of severity), corresponding to 26,408 (67.9%) of the total 38,864 Hb measurements; of these, 20,430 (77.4%) occurred after treatment start (D1-D28). Moderate or more severe anaemia (≥grade 2) occurred in 47.6% of all measurements (18,486/38,864), in 76.5% of episodes (14,148/18,486) after treatment start (D1-D28). Post-D0 anaemia was less frequent on ACT: all-grade (77.3% vs. 80.3%, *p* = 0.036); ≥grade 2 anaemia (76.4% vs. 81.1%, *p* = 0.007).

#### Severity of anaemia

Five patients had very severe anaemia (grade 4) at D0. Overall, 19 patients had 27 records of transient very severe anaemia, corresponding to 2/1000 patients (19/8897). All episodes of very severe anaemia occurred by D14 in ACT groups, corresponding to 1 per 1000 (27/26,408) of all anaemia episodes, with no difference between treatments. Only one patient had very severe anaemia on D28 concomitant with recurrent infection, while all other patients recovered after D14. No case of very severe anaemia was observed in patients treated with non-ACT.

#### Changes in anaemia status

Of the 8720 patients with at least one additional post-D0 Hb measurement, 67.9% were anaemic on presentation and 47.1% when last seen – the latter was composed of 41.9% who remained anaemic plus an additional 5.2% who became anaemic post-D0. Anaemia grade worsened in 31.9% patients.

Mixed-effect multivariate logistic regression model showed an increased risk of anaemia worsening in patients with higher D0 parasitaemia (AOR 1.65, 95% CI 1.52–1.79, *p* = 0.001) and Hb (AOR 1.57, 95% CI 1.51–1.63, *p* = 0.001), younger age (AOR 0.95, 95% CI 0.94–0.97, *p* = 0.001, per year of age). It detected no significant association with parasitological failure (AOR 0.94, 95% CI 0.81–1.12, *p* = 0.452) and no difference between treatments, except a lower risk with AQ + SP (AOR 0.51, 95% CI 0.36–0.72, *p* = 0.001) compared to ASAQ.

### Haemoglobin levels during treatment and follow-up

#### Haemoglobin levels changes (D0 – D3 – D28)

Ten countries and 15 sites (Burkina Faso, Gabon, Liberia, Mali, Mozambique, Nigeria, Rwanda, Uganda, Zambia, Zanzibar) had Hb measurements on D0, D3 and then weekly from D7-D28 (*n* = 4886 inclusive of the 1645 above; median age 2 years, range 6 months to 14 years). Between D0 and D28, Hb increased by +13.8% (from 9.4 to 10.7 g/dl).

#### Haemoglobin levels changes between D0 and D3

On D3, Hb concentrations decreased −4.6% (95% CI -4.3% to −4.9%), from 9.4 to 9.0 g/dl; such decrease was of −4.5% (95% CI −.2% to −4.8%), from 9.3 to 8.9 g/dl, in patients treated with ACT and −7% (95% CI -8.5%-5.6%) on AS. The D3 drop in Hb was greater for subjects with normal D0 Hb (from 11.9 to 10.8 g/dl, −9.3%, 95% CI -9.9% to −8.6%) than for those who were anaemic (from 8.9 to 8.6 g/dl, −3.4%, 95% CI -3.7% to −3.0%, *p* = 0.001).

A multivariate logistic regression model with mixed-effect showed that the risk was higher for patients with higher D0 parasitaemia (AOR 2.51, 95% CI 2.23–2.83, *p* = 0.001) and Hb (AOR 1.19, 95% CI 1.14–1.24, *p* = 0.001) and lower for older subjects (AOR 0.87, 95% CI 0.83–0.92, *p* = 0.001), while no significant association was found with parasitological failure (AOR 1.13, 95% CI 0.95–1.34, *p* = 0.177) and treatment (all comparisons *p* > 0.1).

#### Time to haemoglobin nadir, children <5 years-old

Two studies enrolled 1645 children <5 years-old (median age 2.3 years, range 0.5–4.9) in Liberia [19], Mozambique, Rwanda, and Uganda [13] and recorded Hb on D0-D3, D7, D14, D21, D28. D0 parasite density was 23,908 /μl overall but varied across sites (*p* = 0.001). Median time-to-nadir (Tn) was D2 Table 2), and was independent of treatment (all comparisons *p* > 0.1). The predicted median T_n_ calculated by linear regression was 35 h, ranging from 29 h with ASAQ in Uganda to 48 h with DP in Rwanda (Additional file 6: Fig. S3).

The nadir occurred in 78% between D1–3, with the highest proportion on D2 (34%). The highest rate of Hb decrease (−12.6%) and slope (−1.25) were at D1. The PRR_d1_ was highest (98.4%) for the patients whose Hb increased post-D0 (HDR_d1_ + 8.3%) and lowest (83.8%) in those with nadir at D3 (HDR_d1_–1.8%). Excluding the 11% subjects with no decrease, overall Hb dropped from 9.8 g/dl to 8.7 g/dl (−1.18 g/dl or - 11.4%, 95% CI -11.0% to −11.8%, *p* = 0.001) (Table 2). The patterns of evolution of Hb levels are shown in Additional file 7: Fig. S4.

**Fig. 4:**
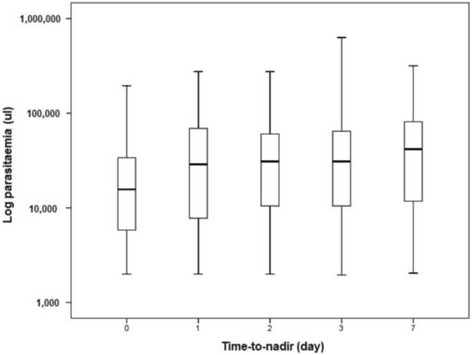
Parasite density pre-treatment (D0) and time-to-nadir, children under 5 years old (Liberia, Mozambique, Rwanda, Uganda; *n* = 1645)

Subjects whose Hb increased after baseline (nadir on D0) had significantly lower D0 parasitaemia (14,410 μl) and Hb (9.0 g/dl) than those whose Hb decreased post-D0 (parasite density = 25,403/μl; Hb = 9.9 g/dl, *p* = 0.001). Subjects with higher D0 parasitaemia had longer T_n_ (*r* = 0.281, *p* = 0.001, Spearman test); the correlation between parasitaemia and T_n_ occurring between D1 and D7 was also significant (*r* = 0.060, *p* = 0.021, Spearman test) (Fig. 4). T_n_ was longer, and the drop in Hb greater, in patients without D0 anaemia (mean T_n_ 3.0 days; Hb change −14.7) than those presenting with anaemia (mean T_n_ 2.1 days; Hb change −10.4%, *p* = 0.001). The PCT (but not PRRd1) of patients whose Hb increased right after treatment start was significantly faster (*p* = 0.001) than that of patients with a decrease in Hb (whatever the time to nadir); no difference was apparent for either parameters between the groups with shorter and longer time to nadir.

Different approaches using linear regression mixed-effect multivariate analyses produced consistent results, whether the model includes the PRR_d1_, PCT or HDR_d1_. The Hb percentage decrease at T_n_ was greater in subject with higher D0 Hb and parasitaemia (*p* = 0.001) and those with longer PCT (*p* = 0.001), and smaller in those with higher PRR_d1_. A slight difference between models was that the Hb percentage decrease was independent of age when PRR_d1_ is used in the model (*p* = 0.088) but lower in older patients (*p* = 0.010) when using PCT. It was lower in patients treated with AL or DP than ASAQ (*p* = 0.001 in all comparison except DP when using PCT, *p* = 0.070).

Special attention was paid to the interaction between Hb and parasitaemia at D1 (when on average ~ 93% of the parasites were cleared – see Table 2). The rate at which Hb fell (measured as Hb decrease rate from D0 to D1, HDR_d1_) was slower in patients with longer T_n_ and faster in patients with higher D0 Hb (*p* = 0.001). Concerning parasitaemia, HDR_d1_ was higher in patients with higher D0 parasitaemia (*p* = 0.001), and those with longer PCT (*p* = 0.001), but was unrelated to PRR_d1_ (*p* = 0.112) and age (*p* = 0.296 and *p* = 0.124 in either models). HDR_d1_ was less in patients treated with AL or DP than with ASAQ (*p* = 0.001).

#### Haemoglobin recovery post-treatment

**Fig. 5:**
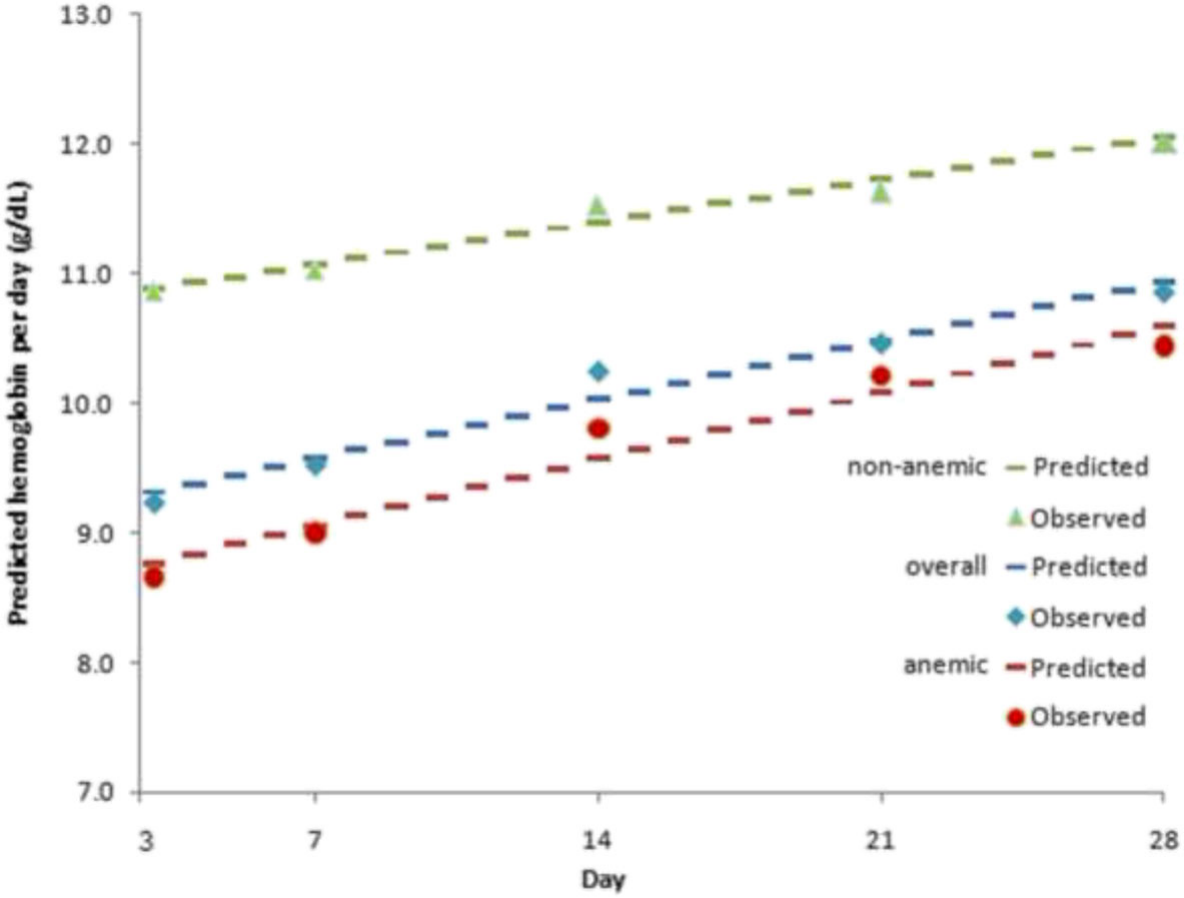
Day 3 to Day 28, overall predicted trend and observed haemoglobin values according to anaemia status on admission, children under 15 years old (Burkina Faso, Gabon, Liberia, Mali, Mozambique, Nigeria, Rwanda, Uganda, Zambia, Zanzibar; *n* = 4886) EquationsOverall: y = 0.065× + 9.1, *p* = 0.001By anaemia status:• anaemia on D0 y = 0.073× + 8.5, *p* = 0.001• no anaemia on D0 y = 0.047× + 10.4, *p* = 0.001By age (not shown):• under 5’s y = 0.71× + 8.8, *p* = 0.001

After an initial decrease in Hb concentrations, a + 19.4% increase from D3 to D28 (from 8.9 to 10.7 g/dl) was observed with a steady significant linear increment in Hb of +0.065 g/dl/day throughout D28, i.e. +0.6% daily (Fig. 5). Patients with normal D0 Hb levels experienced a significant slower recovery (+0.047 vs. +0.073 g/dl/day, *p* = 0.001; corresponding to a daily Hb increase of +0.4% and +0.7%, respectively) than those with pre-treatment anaemia.

Linear regression mixed-effect model found that the recovery in under 5’s was not significantly different from the 5–15 years-old (*p* = 0.059), but that it was significantly faster in patients with anaemia on D0 than those without anaemia (*p* = 0.001). No between-treatment difference was detected.

## Discussion

While anaemia is a common feature of uncomplicated falciparum malaria, studying anaemia during and after malaria treatment is challenging because it is difficult to disentangle the contributions of the infection itself, the process of recovering from the disease, and drug-induced adverse events. Furthermore, Hb is measured inconsistently across antimalarial drug trials during treatment and follow-up, and sometimes not often enough to describe with sufficient precision the patterns of Hb changes. Lastly, the definition and grades of anaemia may vary across studies. All this calls for more standardised study methodologies.

This analysis, representing a wide range of different geographical areas of Sub-Saharan Africa (nine studies at 24 sites in 13 countries), concerns nearly 9000 patients (of whom three-quarters are children under 5 years of age) treated with either ACT or non-ACT for uncomplicated falciparum malaria. Although the frequency of sampling during the study varied, Hb changes resulting in anaemia could be studied during treatment and follow-up and could be compared between treatments. Data were analysed both as continuous (Hb concentration) and categorical variable (age-dependent presence and grade of anaemia [22]), and risk factors for anaemia and drop in Hb concentrations were assessed.

### Anaemia risk on presentation with an acute uncomplicated falciparum malaria episode

Anaemia was common in this largely paediatric patient population: over two-thirds presented with anaemia, and as many as 83% had at least one on-study Hb value below the anaemia-defining cut-off.

Comparing these results between this dataset and other published studies is difficult, as different cut-offs were used to define anaemia and some studies report mean or median Hb levels but not anaemia prevalence. Drawing generalizable conclusions is further complicated by the absence of information on background intensity of transmission and other conditions which may cause Hb to fall. For instance, considering recent studies, using Hb <11 g/dl as the general cut-off, the prevalence of anaemia was 36% in Mali in all-ages subjects with acute malaria (*n* = 5990) with a geometric mean of ~18,000 parasites/μl [23]. When using haematocrit <30% (~Hb <10 g/dl) in a cohort of 672 Nigerian <5’s with acute malaria, anaemia was found in 44% of subjects [24]. Other studies report median Hb = 8.6 g/dl (>5000 children <11 years-old, mean parasitaemia 9500 parasites/μl in Gabon) [25]; mean Hb = 10.6 g/dl (>900 children <6 years-old, mean parasitaemia ~40,000 parasites/μl in Mali) [26]; mean Hb =0.7 g/dl (~800 children <10 years-old, mean parasitaemia 1250 parasites/μl in Zambia) [27]; mean Hb = 9.9 g/dl - 10.4 g/dl (children <11 years-old, median parasitaemia ~14,000 parasites/μl in Cameroon) [28]. Even within the present analysis we found wide inter-country variations, with pre-treatment prevalence of anaemia ranging from 2.3% (in Liberia where patients had lower parasitaemia [19]) to 80.1% in Zanzibar, [21], and mean pre-treatment Hb from 8.6 g/dl, Zanzibar [21] to 12.9 g/dl in Liberia [19].

While malaria is known to cause anaemia, the extent of Hb loss caused by each individual episode in not well quantified. In a previous paper [29] we estimated this drop to average 1.2 g/dl or 13% of a subject “normal status”.

Not unexpectedly, young age was found to be a risk factor for anaemia on presentation with acute falciparum malaria: compared to circa three-quarters of the under 5’s presented with anaemia, the relative risk was reduced by half to two-thirds in older subjects. There was a significant inverse correlation between parasitaemia and Hb concentrations overall.

### The fall and rise of haemoglobin

Hb drops soon after treatment starts to reach its nadir (−4% to −6%) between D1-D3, thus supporting the use of the latter as proxy for maximum Hb fall in the standard clinical trial. Thereafter Hb increases at a steady rate of ca. 0.6% per day. Patients with normal D0 Hb levels experience a greater fall (−6% vs. -3.1%) and slower recovery ((+0.4% and +0.7% per day, respectively) than those with pre-treatment anaemia. This study found no evidence of delayed anaemia associated with oral artemisinin derivatives, or any other treatment; in fact, the risk of anaemia decreased with follow-up time.

This general trend hides three patterns of evolution of Hb concentrations which become apparent in under 5’s undergoing intense monitoring: (a) the dominant response (~78%) is a decrease with the lowest Hb level recorded between D1 and D3. This appears to be a fairly homogeneous population with D0 parasitaemia ~25,000 parasites/μl, PCT ~30 h [30], Hb 9.7–9.9 mg/dl, and drop at nadir ~ −11.1 to −13.4%; (b) an additional 12% had their Hb nadir at their D7 visit; they had higher pre-treatment parasite counts (~30,000) and Hb (10.7 g/dl) but their final Hb drop was only marginally greater (−13.8%); (c) in a further 11% of these patients Hb started climbing immediately after treatment commenced (+8.3% on D1); they had lower D0 parasite counts and Hb than those whose Hb fell after treatment start. So, both pre-treatment Hb and parasite counts were (b) > (a) > (c), whereas the final Hb concentrations were (c) > (a) > (b). Patients whose Hb started to increase immediately with treatment cleared their parasites faster: the PCT was (c) > (a) = (b).

How are these different patterns explained, and how could they help us understand the relative effects on Hb of uncomplicated falciparum malaria and antimalarial treatment? We do not have all the elements to help us draw definitive conclusions. For instance we do not know how long had the patients been infected, and what the time-course of their Hb level had been before they came into observation. We know that malaria-induced anaemia is accounted for not only by the destruction of parasitized RBCs but also by the removal of uninfected RBCs and by central effects on erythropoiesis. If already on a downwards trend before treatment, Hb is therefore expected to continue to fall because some of these events will continue even when parasites are being killed by the treatment. In fact, parasite killing accounts for a small proportion of the Hb loss. Take for instance patients whose nadir was on D1 (D0 parasitaemia = 23,221/μl; PRR_d1_ = 95.4%, HDR_d1_–12.6%, Hb drop −1.27 g/dl), and assuming the MCH (mean cell Hb) to be 25 picograms (=2.5^−11^ g/RBC), a PRR_d1_ = 95.4% would translate in a loss of 2.229 RBCs/dl and 0.055 Hb g/dl. This means that 96% of the total 1.27 g/dl Hb drop is unaccounted for by parasite killing (RBC destruction); this difference would remain very large even after allowing for the biomass of sequestered parasites which are not seen in the peripheral circulation. The total loss in RBCs (as calculated from the drop in Hb) is approximately 20 times that accounted for by parasite clearance. The early Hb drop and the absence of delayed anaemia reported in cases of severe malaria treated with artesunate [31], makes the contribution of ‘pitting’ (removal of ‘once-infected erythrocytes’ by the spleen) in the case of uncomplicated malaria unlikely or unimportant, possibly because of the relatively lower parasite counts in uncomplicated malaria.

The malaria parasite is likely to determine the presence and degree of anaemia both directly by the abovementioned central and peripheral parasite effects, and also indirectly for example by inducing, through generalised vasodilation and internal fluid redistribution, complex changes in fluid balance resulting in the reduction of the effective circulating blood volume [32], which are corrected during recovery. It is therefore possible that, since what we measure is concentrations and not absolute numbers, we have an incomplete picture of the real situation in terms of both anaemia on presentation and recovery post-treatment.

Our analyses point to a correlation between parasite clearance and changes in Hb levels. Linear regression mixed-effect multivariate analysis using different approaches concur to show that the degree of Hb loss after treatment start is greater in patients with high pre-treatment Hb and parasitaemia, is overall unrelated to age, whether measured at D1 as Hb decrease rate (HDR_d1_, approximately 24 h after treatment started) or at the time of nadir as percentage decrease from pre-treatment values. The drop in Hb levels is greater in patients with slower parasite clearance and it is smaller as percent reduction at nadir in those with higher parasite clearance on D1. The early Hb fall seems therefore to be more a question of slower onset of drug action than it is of drug toxicity, is a short-term event, and is independent of treatment ultimately succeeding or failing or of patient’s age.

## Conclusions

This study clarifies issues about risk factors of anaemia in uncomplicated falciparum malaria before and after treatment. It identifies risk factors on presentation (young age, high parasitaemia), detects different patterns of responses following antimalarial treatment (immediate increase, or early or late decline followed by a linear increase afterwards to restore normal Hb concentrations), shows that the majority of subjects experience a drop within 1–2 days (thus earlier than commonly thought), and finds a relationship between the degree of the early Hb dip and slower parasite clearance. However, we still have an incomplete picture of the overall interaction between malaria infection, antimalarial treatment and anaemia risk, especially when it comes to the explaining the complex relationship between infection, treatment and haemoglobin.

## Additional files

Additional file 1: Table S1. Anaemia grades (g/dl) of severity according to age and sex. (DOCX 14 kb)

Additional file 2: Table S2. Breakdown by treatment. (DOCX 14 kb)

Additional file 3: Figure S1. Number of patients by treatment. AS, artesunate; AQ, amodiaquine; ASAQ, artesunate-amodiaquine; AL, artemether-lumefantrine; DP, dihydroartemisinin-piperaquine; SP, sulphadoxine-pyrimethamine. (TIFF 1276 kb)

Additional file 4: Table S3. Study characteristics: number of Hb measurements (total and by treatment) and measurement days. (DOCX 17 kb)

Additional file 5: Figure S2. Scatter plot of haemoglobin concentration values and parasitaemia pre-treatment (D0): overall and by age and sex category. For the whole study population the equation calculated by linear regression with mixed effect model was y = − 0.06× + 10.3, *p* = 0.020 (overall), y = 0.08× + 9.30, *p* = 0.021 (children <5 years-old) and y = − 0.21× + 12.4, *p* = 0.001 (subjects of 5 years of age or more). (TIFF 4286 kb)

Additional file 6: Figure S3. Predicted median time-to-nadir by country and treatment group, children under 5 years of age (Liberia, Mozambique, Rwanda, Uganda; *n* = 1645). ASAQ, artesunate-amodiaquine; AL, artemether-lumefantrine; DP, dihydroartemisinin-piperaquine; Parasite density was expressed in geometric mean (μl). (TIFF 2364 kb)

Additional file 7: Figure S4. Mean haemoglobin values according to each patient’s anaemia status on admission, children under 5 years old (Liberia, Mozambique, Rwanda, Uganda; *n* = 1645). (TIFF 1913 kb)

## Abbreviations

ACT: Artemisinin combination therapy
AL: Artemether-lumefantrine
AOR: Adjusted risks
AQ: Amodiaquine
AS: Artesunate
AS + SP: Artesunate plus sulphadoxine/pyrimethamine
CI: Confidence intervals
DP: Dihydroartemisinin-piperaquine
Hb: Haemoglobin
HDRd1: Haemoglobin decrease rate by Day 1
IPD: Individual participant-level database
PCT: Parasite clearance time
PRRd1: Parasite reduction rate by Day 1
RCT: Randomized controlled trials; T_n_, time-to-nadir
