# Supplementary material for: Haemoglobin changes and risk of anaemia following treatment for uncomplicated falciparum malaria in sub-Saharan Africa
Source: BMC Infect Dis. 2017 Jun 23;17:443. doi: 10.1186/s12879-017-2530-6 (PMC5481927; doi:10.1186/s12879-017-2530-6)
Supplement: Supplementary file 1 — Anaemia grades (g/dl) of severity according to age and sex. (DOCX 14 kb) [file 12879_2017_2530_MOESM1_ESM.docx]

Table S1: Anaemia grades (g/dl) of severity according to age and sex

| Age group |  |  | mild | moderate | severe | very severe |
| --- | --- | --- | --- | --- | --- | --- |
|  |  |  | grade 1 | grade 2 | grade 3 | grade 4 |
| Children 6 - 59 months | | | <11.0-10.0 | <10.0 - 7.0 | <7.0 - 5.0 | <5.0 |
| Children 5 - 11 years of age | | | <11.5-11.0 | <11.0 - 8.0 | <8.0 - 5.0 | <5.0 |
| Children 12 - 14 years of age | | | <12.0-11.0 | <11.0 - 8.0 | <8.0 - 5.0 | <5.0 |
| Women, non-pregnant  (15 years of age and above) | | | <12.0-11.0 | <11.0 - 8.0 | <8.0 - 5.0 | <5.0 |
| Men (15 years of age and above) | | | <13.0-11.0 | <11.0 - 8.0 | <8.0 - 5.0 | <5.0 |
