# Supplementary material for: Haemoglobin changes and risk of anaemia following treatment for uncomplicated falciparum malaria in sub-Saharan Africa
Source: BMC Infect Dis. 2017 Jun 23;17:443. doi: 10.1186/s12879-017-2530-6 (PMC5481927; doi:10.1186/s12879-017-2530-6)
Supplement: Supplementary file 2 — Breakdown by treatment. (DOCX 14 kb) [file 12879_2017_2530_MOESM2_ESM.docx]

Table S2: Breakdown by treatment

| Treatment |  | N | % |
| --- | --- | --- | --- |
| Artemisinin  Combination  Therapy (ACT) | *All ACTs* | 7,968 | 89.6% |
|  | artesunate-amodiaquine, ASAQ fixed-dose | 2,280 | 25.6% |
|  | artesunate-amodiaquine, ASAQ loose or co-blister | 1,106 | 12.4% |
|  | artemether-lumefantrine, AL | 2,609 | 29.3% |
|  | artesunate plus sulphadoxine/pyrimethamine, AS+SP | 249 | 2.8% |
|  | dihydroartemisinin-piperaquine, DP | 1,724 | 19.4% |
| non-ACT | *All monotherapies* | 402 | 4.5% |
|  | amodiaquine alone, AQ | 150 | 1.7% |
|  | artesunate alone, AS | 252 | 2.8% |
|  | *All non-artemisinin containing combinations* | 527 | 5.9% |
|  | amodiaquine plus sulphadoxine/pyrimethamine, AQ+SP | 527 | 5.9% |
| Total |  | 8,897 | 100.0% |
|  |  |  |  |
