# Supplementary figures and images for: Haemoglobin changes and risk of anaemia following treatment for uncomplicated falciparum malaria in sub-Saharan Africa
Source: BMC Infect Dis. 2017 Jun 23;17:443. doi: 10.1186/s12879-017-2530-6 (PMC5481927; doi:10.1186/s12879-017-2530-6)

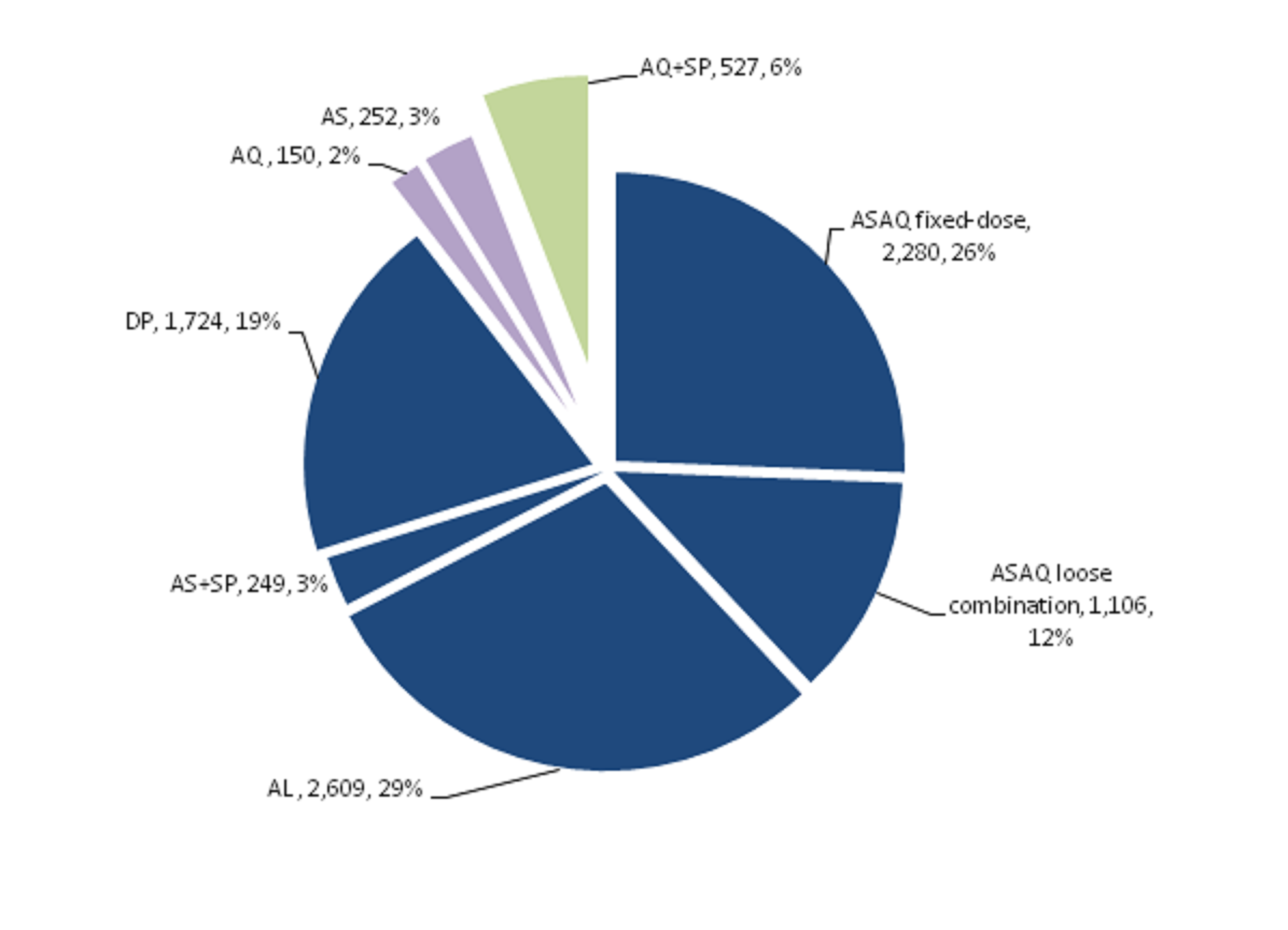

Supplement: Supplementary file 3 — Number of patients by treatment. AS, artesunate; AQ, amodiaquine; ASAQ, artesunate-amodiaquine; AL, artemether-lumefantrine; DP, dihydroartemisinin-piperaquine; SP, sulphadoxine-pyrimethamine. (TIFF 1276 kb) [file 12879_2017_2530_MOESM3_ESM.tif]

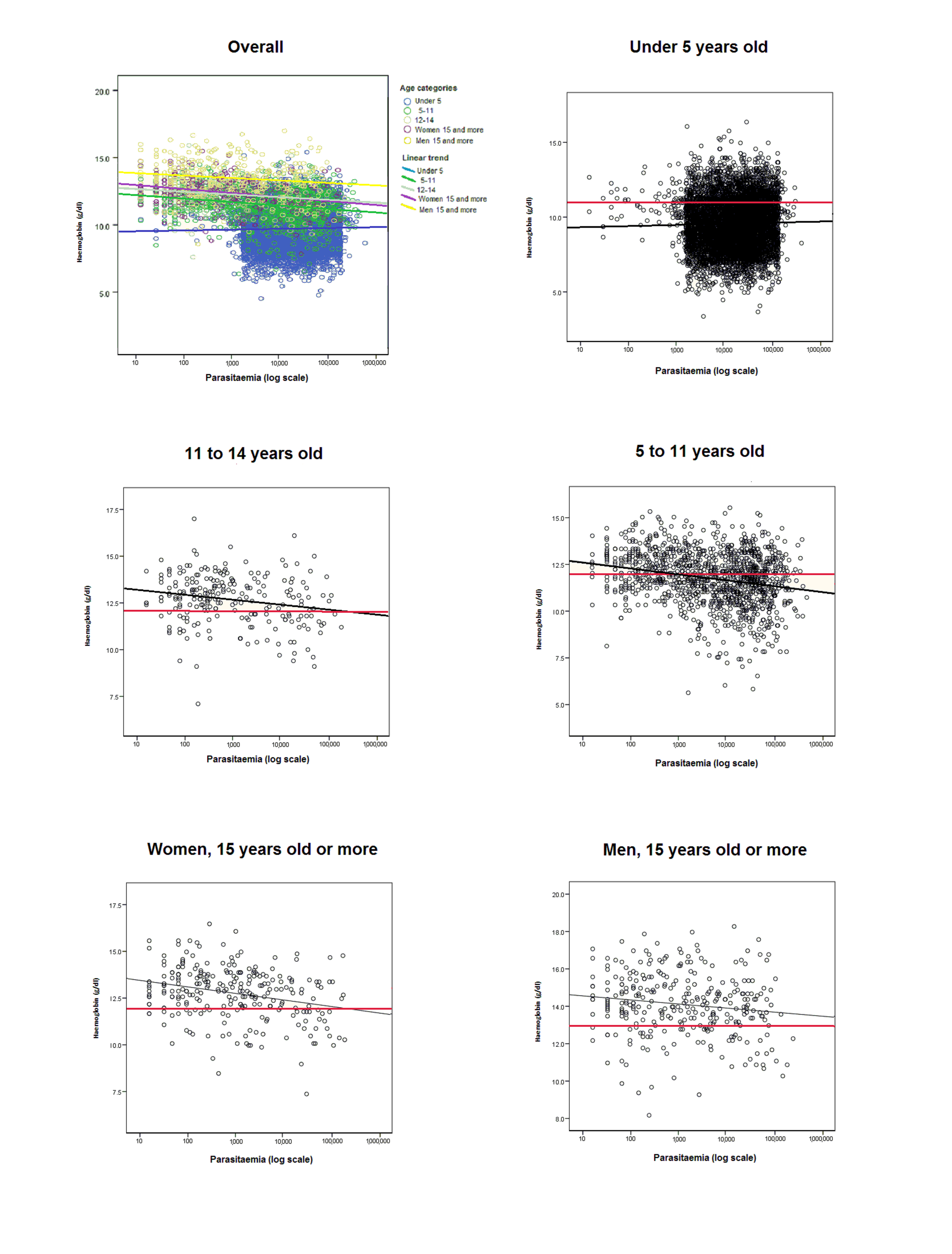

Supplement: Supplementary file 5 — Scatter plot of haemoglobin concentration values and parasitaemia pre-treatment (D0): overall and by age and sex category. For the whole study population the equation calculated by linear regression with mixed effect model was y = − 0.06× + 10.3, p = 0.020 (overall), y = 0.08× + 9.30, p = 0.021 (children <5 years-old) and y = − 0.21× + 12.4, p = 0.001 (subjects of 5 years of age or more). (TIFF 4286 kb) [file 12879_2017_2530_MOESM5_ESM.tif]

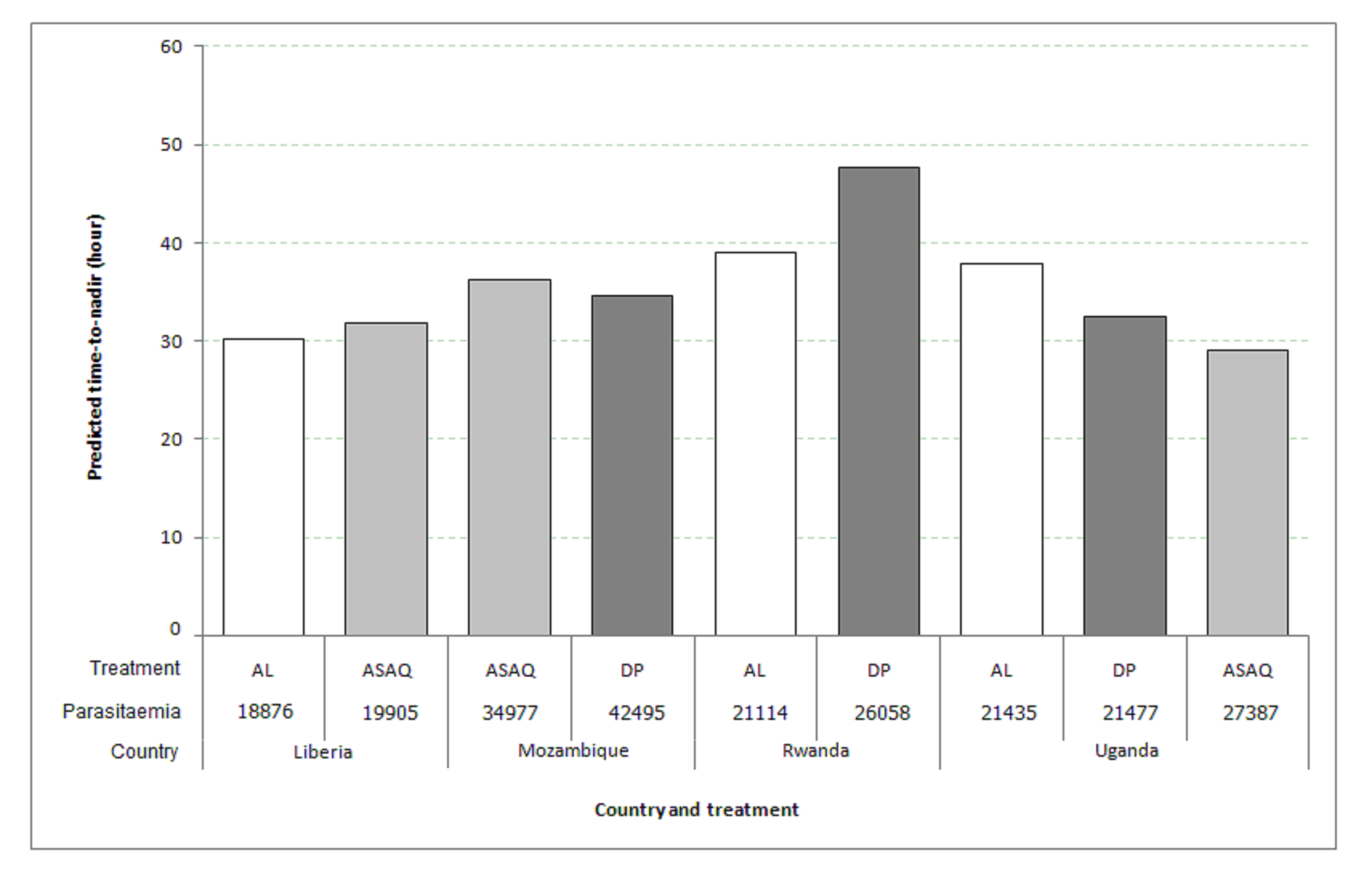

Supplement: Supplementary file 6 — Predicted median time-to-nadir by country and treatment group, children under 5 years of age (Liberia, Mozambique, Rwanda, Uganda; n = 1645). ASAQ, artesunate-amodiaquine; AL, artemether-lumefantrine; DP, dihydroartemisinin-piperaquine; Parasite density was expressed in geometric mean (μl). (TIFF 2364 kb) [file 12879_2017_2530_MOESM6_ESM.tif]

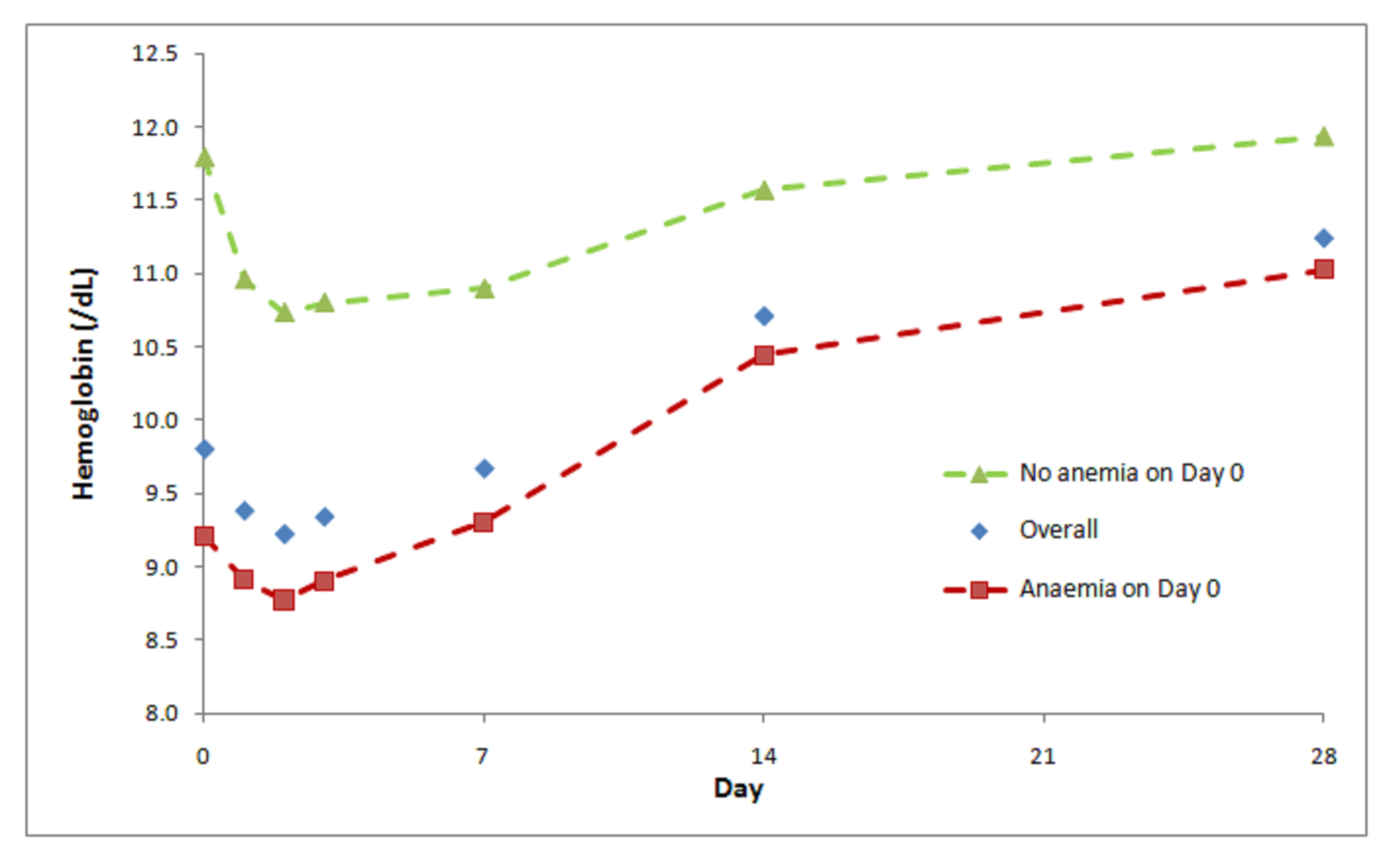

Supplement: Supplementary file 7 — Mean haemoglobin values according to each patient’s anaemia status on admission, children under 5 years old (Liberia, Mozambique, Rwanda, Uganda; n = 1645). (TIFF 1913 kb) [file 12879_2017_2530_MOESM7_ESM.tif]
