# Supplementary material for: Haemoglobin changes and risk of anaemia following treatment for uncomplicated falciparum malaria in sub-Saharan Africa
Source: BMC Infect Dis. 2017 Jun 23;17:443. doi: 10.1186/s12879-017-2530-6 (PMC5481927; doi:10.1186/s12879-017-2530-6)
Supplement: Supplementary file 4 — Study characteristics: number of Hb measurements (total and by treatment) and measurement days. (DOCX 17 kb) [file 12879_2017_2530_MOESM4_ESM.docx]

Table S3: Study characteristics: number of Hb measurements (total and by treatment) and measurement days

|  | Patients | Sites | Measurements | ACT | | | | non-ACT |  |
| --- | --- | --- | --- | --- | --- | --- | --- | --- | --- |
| Country [reference] | N | N | N | ASAQ | AL | DP | AS+SP |  | Days of measurement |
| Burkina Faso [13] | 810 | 1 | 3,854 | 36% | 36% | 27% |  |  | D0, D3, D7, D14, D28 |
| Cameroon [17] | 166 | 1 | 441 | 66% | 34% |  |  |  | D0, D7, D28 |
| Gabon [13] | 226 | 1 | 1,044 | 35% | 35% | 30% |  |  | D0, D3, D7, D14, D28 |
| Liberia [19] | 1000 | 1 | 2,331 | 50% | 50% |  |  |  | D0, D1, D2, D7, D28 |
| Liberia [20] | 300 | 1 | 4,883 | 50% | 50% |  |  |  | D0, D1, D2, D3, D7, D14, D21, D28, D35, D42 |
| Madagascar [17] | 179 | 1 | 523 | 66% | 34% |  |  |  | D0, D7, D28 |
| Mali [14] | 753 | 1 | 549 | 33% |  |  | 33% | 33% (AS) | D0, D3, D7, D14, D21, D28 |
| Mali [17] | 203 | 1 | 3,693 | 67% | 33% |  |  |  | D0, D7, D28 |
| Mozambique [13] | 420 | 1 | 2,302 |  | 50% | 50% |  |  | D0, D1, D2, D3, D7, D14, D28 |
| Nigeria [13] | 494 | 2 | 1,736 | 35% | 35% | 30% |  |  | D0, D3, D7, D14, D28 |
| Rwanda [16,18] | 1070 | 4 | 2,058 | 38% |  | 23% |  | 38% (AQ) | D0, D14, D28 |
| Rwanda [13] | 295 | 3 | 2,267 |  | 50% | 50% |  |  | D0, D1, D2, D3, D7, D14, D28 |
| Senegal [17] | 392 | 1 | 1,107 | 67% | 33% |  |  |  | D0, D7, D28 |
| Uganda [13] | 1212 | 3 | 7,379 | 13% | 37% | 50% |  |  | D0, D1, D2, D3, D7, D14, D28 |
| Uganda [15] | 730 | 1 | 1,430 | 33% |  | 30% |  | 37% (AQ+SP) | D0, D14 |
| Zambia [13] | 245 | 1 | 878 | 35% | 34% | 31% |  |  | D0, D3, D7, D28 |
| Zanzibar [21] | 402 | 2 | 2,389 | 50% | 50% |  |  |  | D0, D3, D7, D14, D21, D28 |
| Total | 8,897 | 20 | 38,864 | 38% | 29% | 22% |  | 10% |  |

Legend: ACT, artemisinin combination therapy; AS, artesunate; AQ, amodiaquine; ASAQ, artesunate-amodiaquine; AL, artemether-lumefantrine; DP, dihydroartemisinin-piperaquine; SP, sulphadoxine-pyrimethamine;
